# Hunting the Needle in the Haystack: A Guide to Obtain Biologically Meaningful MicroRNA Targets

**DOI:** 10.3390/ijms151120266

**Published:** 2014-11-06

**Authors:** Michael Karbiener, Christina Glantschnig, Marcel Scheideler

**Affiliations:** RNA Biology Group, Institute of Molecular Biotechnology, Graz University of Technology, Petersgasse 14, 8010 Graz, Austria; E-Mails: michael.karbiener@tugraz.at (M.K.); christina.glantschnig@tugraz.at (C.G.)

**Keywords:** small non-coding RNA, microRNA, microRNA–mRNA interaction, direct microRNA target, microRNA target identification, direct microRNA target validation

## Abstract

MicroRNAs (miRNAs) are endogenous small non-coding RNAs of ~23 nucleotides in length that form up a novel class of regulatory determinants, with a large set of target mRNAs postulated for every single miRNA. Thousands of miRNAs have been discovered so far, with hundreds of them shown to govern biological processes with impact on disease. However, very little is known about how they specifically interfere with biological pathways and disease mechanisms. To investigate this interaction, the hunt for direct miRNA targets that mediate the miRNA effects—the “needle in the haystack”—is an essential step. In this review we provide a comprehensive workflow of successfully applied methods starting from the identification of putative miRNA-target pairs, followed by validation of direct miRNA–mRNA interactions, and finally presenting methods that dissect the impact of particular miRNA-target pairs on a biological process or disease. This guide allows the way to be paved for obtaining biologically meaningful miRNA targets.

## 1. Introduction

In 1993, Victor Ambros and co-workers for the first time described a gene coding for small RNAs that are not translated, yet regulate another, protein-coding gene via base-pairing to its mRNA [1]. This seminal study on the *Caenorhabditis elegans* lin-4 RNA and LIN-14 protein already captured some fundamental aspects about the function of microRNAs (miRNAs), *i.e.*, that they negatively affect protein synthesis (at least in most instances), that they bind predominantly within the 3' untranslated region (3' UTR) of mRNAs, and that, in animals, this base-pairing is usually imperfect. However, it took almost a decade to understand the biological importance of this mechanism—later coined RNA interference (RNAi)—in its full depth. First, it was not before 2000 that the second miRNA—let-7—was discovered in *C. elegans* [2]. In contrast to lin-4, let-7 was found to be highly conserved, with orthologs existing in many invertebrate and also vertebrate species, including human [3]. Second, as even the original publication on let-7 described its binding to several distinct mRNAs, the broad spectrum of direct miRNA targets could be anticipated for the first time.

Soon after the discovery of let-7, the existence of many other, similar small RNAs was reported in various species, and the novel class of transcripts was termed “microRNAs” (miRNAs) [4,5,6]. Subsequent research brought to light more and more distinct miRNAs and also elucidated their biogenesis (see [7] for an extensive review): originating from longer primary transcripts, a characteristic “hairpin” secondary structure of the RNA molecule serves as substrate for two sequential endonucleolytic processing steps catalyzed by two distinct protein complexes. Within the first complex, the RNase III enzyme Drosha cleaves both at the 5' and 3' end of the hairpin to release the ~70-nucleotide precursor miRNA (pre-miRNA) [8]. The pre-miRNA is subsequently exported into the cytoplasm (involving the Ran-GTP-dependent cargo transporter Exportin 5) [9], where it is subjected to further trimming within the second complex that harbors the RNase III enzyme Dicer. Dicer cleaves off the hairpin loop, thereby generating two mature miRNAs of ~23 nucleotides in length [10], which are then separated and can both be incorporated in the miRNA-induced silencing complex (miRISC). At the core of this complex, the mature miRNA is physically associated with members of the Argonaute protein family (Ago 1–4 in human) [11], resulting in decreased protein output due to reduced translation, or degradation of the targeted mRNA. Only Ago2 was shown to possess endonucleolytic (“slicer”) activity that leads to miRNA-guided mRNA cleavage (and subsequent degradation) [12] if sequence complementarity between the miRNA and the respective miRNA recognition element (MRE) on the mRNA is high. Apart from “slicing”, several other miRNA-guided processes result in decreased protein output. First, mRNA deadenylation, leading to rapid mRNA decay [13], was found to be mediated by GW182/TNRC6 proteins, further miRISC components that directly interact with Ago proteins and recruit the deadenylase complexes CCR4–CAF1–NOT and PAN2–PAN3 [14,15]. Second, the miRISC can also prevent translation initiation via binding the mRNA’s 5'-7-methylguanosine (m^7^G) cap, thereby precluding the association of the eukaryotic translation initiation factor eIF4E with the mRNA [16]. Physical interaction of GW182/TNRC6 proteins with poly(A)-binding protein (PABP) might likewise hamper the formation of a “closed-loop” structure between the mRNA’s 5' and 3' ends. In addition, joining of the 60S ribosomal subunit to the 40S-mRNA complex might be impeded by miRISC [17,18], again preventing the formation of a full ribosome that is capable of translation. Third, miRNAs also interfere with protein synthesis after initiation of translation [19,20], yet the precise molecular mechanisms of this process remain to be identified. It should be emphasized that the temporal order of translational repression and mRNA decay is still a matter of debate. For instance, two recent reports used elegant experiments in fly and zebrafish to show that translational repression precedes mRNA decay [21,22]. In contrast, a similar study on human cells found that the fraction of decreased protein output attributable to translational repression is generally small, and also remains small when comparing late to early time points after miRNA modulation [23]. Even though the experimental design of this study cannot fully exclude a rapid mRNA destabilization caused by translational repression, it should be noted that some reports have found that mRNA decay also occurs if translation of the respective mRNA is blocked [13,24,25].

While plant miRNAs usually bind to MREs with almost complete sequence complementarity, metazoan miRNAs require less perfect base pairing to interact with an mRNA. As a consequence, the number of potential miRNA–mRNA interactions is comparatively high in animal genomes and the identification of “true” (*i.e.*, biologically functional) interactions is non-trivial. Some fundamental rules that are valid for the majority of miRNA–mRNA interactions were worked out already at the beginning of miRNA research. First, and most important, a region designated as miRNA “seed”, comprising positions 2-7 or 2-8 of the mature miRNA, usually exhibits perfect and contiguous binding to the mRNA’s “seed match” sequence [26,27,28]; Second, the central region of the miRNA-mRNA duplex (positions 9-12 of the mature miRNA) usually contains mismatches; Third, base pairing of the miRNA’s 3' half to the mRNA increases the thermodynamic stability of a miRNA–mRNA duplex and can thereby compensate for imperfect seed to seed match pairing [28,29]; Fourth, miRNAs most frequently bind to the 3' UTR of the mRNA, and for long 3' UTRs, there is a bias of MRE location either at the beginning (*i.e.*, shortly after the stop codon) or the end (*i.e.*, shortly before the poly(A)-tail) [30]; Fifth, features of the mRNA sequence surrounding particular MREs are additional determinants for effective miRNA targeting; these include adenylate-uridylate-rich (AU-rich) regions (favoring miRNA binding) [31] and competitive, intramolecular secondary structures (preventing the miRNA from binding to the mRNA) [32].

Fourteen years after the discovery of the first miRNA in mammals, more than 2500 and 1900 members of this ncRNA class have been identified in human and mouse, respectively (according to miRBase, release 21 [33]). It is now well established that miRNAs directly regulate a substantial amount of protein-coding genes [34], thereby impacting on most if not all biological processes. Fortunately, high-throughput methods developed during the last decades allow the rapid analysis of protein, mRNA, and also miRNA expression profiles of biological states of interest. Furthermore, diverse libraries are available today to screen, in gain- or loss-of-function experiments, essentially the whole “miRnome” for miRNAs that are functional in a certain biological phenomenon. However, the identification of directly targeted mRNAs that are relevant for the particular phenotype caused by the miRNA is still an elaborate task. This review is intended to provide a possible workflow for researchers in mammalian biology to obtain direct miRNA–mRNA interactions that are of biological significance. First, available bioinformatic tools and databases which collect potentially direct or already validated direct miRNA targets will be discussed. Second, it will be outlined how high-throughput and low-throughput experiments—in combination with aforementioned *in silico* analyses—can aid in obtaining “high priority” target mRNAs. Third, the methodological possibilities to validate direct miRNA–mRNA interactions will be presented. Finally, strategies by which the biological relevance of individual miRNA–mRNA interactions can be elucidated will be summarized.

## 2. Identification of Putative Direct MicroRNAs (miRNAs)–mRNA Interactions of Interest

### 2.1. Bioinformatic Tools Predicting Direct miRNA–mRNA Interactions in Mammals

As the combinatorial possibilities for imperfect hybridizations between a mature miRNA of ~23 nucleotides and a 3' UTR of hundreds to thousands of nucleotides are essentially indefinite, the demand for bioinformatic tools was already apparent at the beginnings of miRNA research. A powerful miRNA target prediction should therefore present a list of highly confident miRNA–MRE pairs, *i.e.*, include as few false positives as possible, but simultaneously should not discard any true miRNA–MRE interaction (false negatives). Fortunately, a number of “target-prediction tools” has been generated during the last decade (for an overview of the most frequently used tools, see Table 1). Almost all of them provide a user-friendly web interface enabling the fast query of predicted direct targets for particular miRNAs, or the retrieval of miRNAs predicted to target a particular mRNA. In most cases, also the complete sets of miRNA–mRNA interactions computed by the respective algorithm are available for download.

While the first bioinformatic approaches had a fairly small overlap of jointly predicted direct miRNA–mRNA interactions, this changed when the importance of the seed:seed match pairing was broadly recognized as the prime determinant for miRNA binding [29]. Indeed, all currently available target prediction tools have implemented the search for seed:seed match pairs. However, it is a distinguishing feature between tools how stringently the “seed criterion” is applied: While the algorithms TargetScan [26,31,34,35], PicTar [36,37], ElMMo [30], MirTarget2 [38] miRWalk [39], and miRmap [40] use stringent pairing between seed and seed match, PITA (Probability of Interaction by Target Accessibility) [32], Miranda [41], DIANA-microT [42,43] and RNA22 [44] apply a more relaxed seed criterion that allows for single G:U wobble base pairs within the seed:seed match pairing.

As a second parameter, most target prediction tools compute scores that evaluate the evolutionary conservation of potential seed matches (and their surroundings). As more and more animal genomes were sequenced and aligned, this approach has led to an ever increasing refinement of algorithms [34,35]. It should be noted, though, that this strategy *a priori* excludes the discovery of MREs targeted by species-specific miRNAs, and that many targets might be missed for miRNAs that are poorly conserved across species. In this respect, RNA22 constitutes an alternative to all other algorithms as target site conservation is not considered at all, while PITA and TargetScan predictions, in addition to the (top-ranked) conserved sites, also include predicted sites with poor evolutionary conservation.

Accessibility of mRNA regions for miRISC binding is a further parameter that helps in discerning favorable from less favorable MREs. Obviously, the set of different thermodynamically stable secondary and tertiary structures of an mRNA is likely to “withdraw” MREs from possible interactions with miRNAs. Thus, PITA has implemented calculations estimating the energy needed to unfold mRNA secondary structures, as well as the energy needed to establish miRNA–mRNA binding. Also TargetScan, PicTar, Miranda, RNA22 and MirTarget2 have related approaches that take into account the free energy of miRNA–mRNA duplexes. Moreover, global analysis of miRNA transfection experiments revealed that the vicinity of AU-rich sequences—also rendering the MRE more accessible—has significant positive effects on targeting efficiency [31]. Hence, TargetScan also evaluates each site context in terms of AU content. The importance of target site accessibility has recently been underpinned by Vejnar and Zbodnov, who generated a software library (miRmap) that incorporated and compared 11 distinct prediction parameters (some adopted from TargetScan and PITA). Comparison of this novel *in silico* prediction tool with experimental data from several high-throughput studies (in which individual miRNAs had been modulated and effects on mRNA and protein level had been analyzed) found that site accessibility is indeed the parameter which, on its own, performs best in explaining miRNA-mediated target regulation [40].

**Table 1:** MicroRNA target prediction tools.

Prediction Tool	Criteria for Prediction and Ranking	Last Update	Output Formats	Available Downloads
TargetScan [45]	stringent seed: seed match pairing, number of target sites, free folding energy of miRNA—target site interactions, target site evolutionary conservation, target site context and accessibility	2012	table of miRNA-target interactions, image of 3' UTR with miRNA binding sites, alignment of orthologous 3' UTRs	all target site predictions, algorithms (Perl scripts)
PicTar [46]	stringent seed: seed match pairing, number of target sites, target site evolutionary conservation, predicted optimal free energy of target sites	2007	table of miRNA-target interactions, alignment of orthologous 3' UTRs	all target site predictions
ElMMo [47]	stringent seed: seed match pairing, number of target sites, target site evolutionary conservation	2009	table of miRNA-target interactions, listing of evolutionarily conserved seed matches	all target site predictions
MirTarget2 [48]	stringent seed: seed match pairing, seed match evolutionary conservation, target site base composition, free energy of target site, target site location	2012	table of miRNA-target interactions, 3' UTR sequence with seed matches highlighted	all target site predictions
PITA [49]	seed: seed match paring (G:U wobble base pairs allowed), number of target sites, free folding energy of miRNA—target site interactions and energy cost of 3' UTR secondary structure unfolding	2008	Excel spreadsheets containing miRNA-target interactions for distinct target sites or entire 3' UTRs	all target site predictions, algorithms (Perl scripts)
Miranda [50]	seed : seed match paring (G:U wobble base pairs allowed), number of target sites, target site evolutionary conservation, free energy of miRNA-target duplex	2010	table of miRNA-3' UTR interactions, miRNA-target site alignment	all target site predictions, algorithms (C)
DIANA-microT [51]	seed : seed match paring (G:U wobble base pairs allowed), target site evolutionary conservation	2012	table of miRNA-target interactions, miRNA-target site alignment	all target site predictions
rna22 [52]	seed : seed match paring (G:U wobble base pairs allowed), pattern-based sequence search based on miRNA set, free energy of miRNA-target duplex	2011	table of miRNA interactions with distinct target sites including miRNA-target site alignment	all target site predictions
miRWalk [53]	stringent seed: seed match pairing within 3' UTR, 5' UTR, CDS, and promoter region	2011	table of miRNA-target interactions	predictions for individual miRNAs or targets
miRmap [54]	stringent seed : seed match paring, number of target sites, target site evolutionary conservations, target site context and accessibility, ensemble free energy of miRNA-target interaction	2013	table of miRNA-target interactions, miRNA-target site alignment including scores for every interaction criterion	all target site predictions, algorithms (Python)

Abbreviations: CDS, coding sequence; DIANA, DNA Intelligent Analysis; PITA, Probability of Interaction by Target Accessibility; UTR, Untranslated region.

Despite refining procedures, miRNA-target prediction algorithms can still be expected to contain a considerable fraction of false-positive and an unknown number of false negative results [55]. Thus, when confronted with the task to obtain putative direct targets of a miRNA of interest, the researcher is well-advised to retrieve predictions from not just a single, but several of the distinct target prediction tools. In this respect, miRWalk [39], a comprehensive database listing predictions of eight distinct algorithms, constitutes a time-saving option. The web interface also provides the ability to look for MREs within the 5' UTRs and coding sequences of mRNAs, using the algorithm “miRWalk” (that was jointly launched with the miRWalk database and that relies on strict seed:seed match pairing, yet does not incorporate evolutionary conservation or thermodynamic aspects of miRNA–mRNA binding). This is of interest as, indeed, functional miRNA binding sites have repeatedly been reported in 5' UTRs (at least in some cases resulting in up-regulation of the target protein [56,57]) and coding sequences [58,59], albeit these occur at a significantly lower rate than within 3' UTRs [35].

In stark contrast to the extraordinary dynamics in miRNA research throughout the last decade, refactoring of target prediction web interfaces to implement updates for predictions for newly discovered miRNAs, as well as changes to mRNA sequences, surprisingly, has not been performed on a regular basis. Currently, TargetScan, DIANA-microT, MirTarget2 and miRmap can be expected to provide the most comprehensive predictions for direct miRNA targets, as these tools have been last updated in 2012; all other tools described above have not been revised since 2010 or earlier years. As a consequence, when focusing on a recently discovered miRNA, many available target prediction algorithms might drop out. In this respect, TargetScan, Miranda and PITA offer the possibility to download the code and run the algorithm on a self-selected set of miRNAs. Further, the miRmap web interface offers the possibility to enter a miRNA sequence for which target search will be performed. Alternatively, one can also recapitulate the basic principles of target prediction tools by self-programming in appropriate languages such as PERL (Practical Extraction and Reporting Language) or R: 3' UTRs of organisms can be readily downloaded from NCBI or ENSEMBL webpages, and scripts to identify and count distinct types of seed matches can be considered as low-level computational biology tasks. In order to infer the potential evolutionary conservation of identified seed matches and their surroundings, pre-computed multispecies alignments can be easily queried for the genomic region of interest using the UCSC genome browser. Alternatively, several tools to align sequences of orthologous 3' UTRs are available and easy to use, e.g., MUSCLE (via the European Bioinformatics Institute webpage). Finally, algorithms to calculate RNA secondary structure and free energy of RNA duplexes are amenable via user-friendly web interfaces, e.g., RNAfold [60] or RNAhybrid [61].

### 2.2. Databases that Collect Validated Direct miRNA–mRNA Interactions

Progress in research over the last years has yielded an ever growing number of already validated direct miRNA–target interactions. Correspondingly, some databases have been released that are of high value to obtain, for a miRNA of interest, those MREs which have indeed been proven to be functional, at least in a particular cellular context.

The miRWalk database contains such a compendium of validated direct miRNA targets [39]; however, it must be emphasized that these lists were generated, at least partly, via automated text-mining search of PubMed-listed abstracts and hence contain a considerable fraction of false positive interactions. In contrast, TarBase [62] is a fully manually curated platform that stores validated miRNA–target interactions for a number of mammalian species as well as model organisms. For each interaction, the type of validation experiments that were performed is also recorded. Of note, TarBase has been subjected to repeated updates (the latest conducted in 2012 [63]) since its original release. Lastly, the TarBase information content has been used by Hatzigeorgiou and co-workers to generate the tool miRPath, by which cellular pathways can be identified that are likely affected by individual or multiple miRNAs [64].

Similar to TarBase, the tool miRTarBase [65] has been launched in 2010 as a curated collection of miRNA–target interactions for 17 species. Among other extensions, a recent miRTarBase update [66] has incorporated an “expression profile” feature for distinct miRNA–target pairs: Using public repositories of genomics experiments, the expression levels of the respective miRNA and mRNA are visualized across samples, and the correlation between the miRNA and mRNA is statistically analyzed. Depending on the input dataset (e.g., tissues, disease states), significant anti-correlation between miRNA–target pairs can support the importance of such interactions *in vivo* and also aid in hypothesis development.

A slightly different approach was chosen by Jiang and co-workers, who released miR2Disease in 2009 [67]. This database provides a manually curated compendium in which associations between miRNAs and certain diseases are collected. Additionally, information about validated direct targets is provided that was either obtained from TarBase, or found in the original publication that reported the miRNA-disease association.

To sum up, the above mentioned databases can be considered as valuable resources that should be explored before focusing on individual miRNA–target relationships.

### 2.3. High-Throughput Experimental Methods to Identify Potential Direct Target mRNAs

The nature of most miRNAs is to target a broad set of protein-coding transcripts, estimated to be in the range of 100–200 mRNAs per miRNA [28,36]. Therefore, for any biological phenomenon investigated, the most reasonable strategy to start with is a correspondingly broad type of experimental analysis, *i.e.*, the use of high-throughput approaches. These can be subdivided in methods that analyze either mRNAs or their resulting proteins, as well as in experiments that are conducted with or without alterations of intracellular miRNA levels.

While the first discovered miRNAs (in *C. elegans*) were reported to mainly affect protein levels without degrading mRNAs [1,2], a seminal study by Lim *et al.* showed that transfection of miRNAs in HeLa cells led to significant changes in mRNA expression profiles (measured by microarrays) within 12 h, with a significant enrichment of potential direct (*i.e.*, seed-match-bearing) targets among down-regulated genes [68]. These results were subsequently corroborated by other studies [69,70], and thus, in contrast to earlier contention, a substantial proportion of miRNA effects in mammalian cells is measureable at mRNA level. Indeed, a study on human and mouse cells compared the relative effect of miRNA-mediated mRNA destabilization and miRNA-mediated translational inhibition on decreased protein output, and identified reduced mRNA levels as the predominant factor [23]. Therefore, a first simple experimental strategy is to perform a paired profiling of miRNA and mRNA expression using e.g., microarrays or RNAseq, and then use available datasets on predicted or validated miRNA–mRNA interactions to filter out interesting miRNA–target pairs which exhibit significant anti-correlation across the different samples analyzed. Although successfully applied in the past [71,72], it must be noted that this approach is likely to generate a considerable number of false positive interactions. Microarray analysis of miRNA gain-of-function experiments, as first performed by Lim *et al.* [68], is a superior alternative by which interesting potential direct targets can be identified. While Lim *et al.* still had to design siRNA-like RNA duplexes by themselves to elevate the miRNA of interest, today such “miRNA mimics” can be obtained from several providers. Similarly, pre-miRNA expression constructs have meanwhile become off-the-shelf products. A further reduction of less promising target candidates can be achieved if miRNA over-expression/transfection is complemented with miRNA inhibition/silencing experiments, as for example performed for miR-140 [73]. In the latter case, mRNAs can be considered as highly interesting if they (i) exhibit down-regulation upon miRNA elevation; (ii) exhibit up-regulation upon miRNA depletion; and (iii) are predicted (or already validated) direct targets of the miRNA of interest. As for miRNA loss-of-function experiments, efficient sequestration of mature miRNAs is possible by short antisense oligonucleotides (ASOs) that carry 2’-*O*-methyl modifications or locked nucleic acids (LNAs) [74], which are offered by several companies. Furthermore, “miRNA sponges”, *i.e.*, expression cassettes that generate a transcript with many MREs for the miRNA of interest, offer an alternative method for miRNA loss-of-function experiments [75,76].

A more sophisticated high-throughput approach is based on immunoprecipitation (IP) of Ago proteins to pre-select for transcripts that are miRISC-associated. Strategies include both the use of artificial, epitope-tagged Ago versions [77], as well as high-affinity antibodies against endogenous Ago proteins [78]. After pulldown, the mRNAs associated with the miRISC were originally analyzed via cloning [78], which is nowadays mostly replaced by microarray or next-generation sequencing techniques. Again, a comparative analysis between cells in which the miRNA of interest has been overexpressed/silenced and control cells can yield promising candidates for further investigation. It should be noted that Ago-IP-based methods allow the identification of direct targets regardless of whether they are repressed by mRNA decay or inhibition of translation. However, at least two drawbacks are inherent to the originally described technique. First, artificial mRNA-miRISC associations during cell lysis cannot be ruled out [79]. Second, weak interactions between miRNAs and mRNAs within the miRISC might be lost during the biochemical purification procedure [80]. Both problems have recently been solved by techniques that utilize exposure of cells to UV light in order to crosslink RNA to RNA-binding proteins before proceeding with IP. For instance, high-throughput sequencing of RNA isolated by crosslinking immunoprecipitation (HITS-CLIP) [81] has been successfully applied to map miRNA–mRNA interactions in mouse brain [82]. Briefly, the procedure starts with UV-crosslinking at 254 nm, followed by cell lysis and IP with antibodies directed against Ago. Subsequently, free RNA (*i.e.*, not incorporated in RNA-protein complexes) is degraded by RNase treatment, and the remaining intact RNA fragments are purified (by SDS-PAGE and radioactive labeling) and ligated to 5'- and 3'-adapter sequences to facilitate reverse transcription and deep sequencing. Of note, HITS-CLIP enables the identification of miRNA binding regions within mRNAs [80]. An alternative procedure was published by Hafner *et al.*, coined photoactivatable-ribonucleoside-enhanced crosslinking and immunoprecipitation (PAR-CLIP) [83]. This method exposes the cells to the photoactivatable nucleoside analogon 4-thiouridine (4SU), which is randomly incorporated into RNA during transcription. Subsequent UV light treatment (at 365 nm) results in a much more efficient coupling of RNA to proteins compared to HITS-CLIP. Furthermore, 4SU treatment leads to transitions from A to C in subsequent deep sequencing reads, and the propensity for these transitions was found to be significantly higher in crosslinked compared to non-crosslinked RNA [83]. In subsequent bioinformatic analysis procedures of deep sequencing data, this characteristic can therefore be exploited to identify precisely the MREs within distinct transcripts.

Two-dimensional difference gel electrophoresis (2D-DIGE) has been widely used to identify proteins that differ in abundance between a pair of biological conditions. Briefly, the proteins of two samples are labeled with different fluorophores before separation according to isoelectric point (first dimension) and size (second dimension). Spots with different fluorescence intensities are subsequently excised and analyzed by mass spectrometry. With respect to miRNA research, the straightforward approach is to compare cells in which the miRNA of interest has been overexpressed or silenced to suitable control cells (e.g., transfected with a non-targeting control oligonucleotide, or transduced with a construct expressing a scrambled small RNA). For instance, such an experimental strategy has been successfully applied to identify a direct target of miR-21 in the context of cancer [84].

Analogous to microarray experiments that seek differences in gene expression between two conditions on a global scale, stable isotope labeling with amino acids in cell culture (SILAC) is a powerful technique to obtain such differences at the protein level. Therefore, the cells of interest are cultivated with amino acids that are labeled with different stable (*i.e.*, nonradioactive) isotopes. Subsequent analysis is performed by liquid chromatography-tandem mass spectrometry (LC-MS), where the measured peptides can be assorted to either of two samples as a consequence of the differential labeling. SILAC-based experiments enable a reliable quantification of even small changes in protein abundance, and therefore are highly suited to conduct a proteomic screen for direct miRNA targets [85]. Indeed, the scientific consensus that most miRNAs have modest, yet widespread direct effects on protein synthesis is based substantially on studies involving SILAC [69,70].

Altogether, the researcher is well-advised to perform one of the high-throughput assays outlined above. Combining generated experimental data with pre-existing information on predicted or validated miRNA targets most probably yields a number of interesting candidate mRNAs/proteins for further investigation.

### 2.4. Experimental Methods to Identify Individual Potential Direct Target mRNAs

Regardless of whether putative miRNA–target pairs were obtained just by *in silico* analyses, or in combination with high-throughput experiments, several further analyses should be conducted in order to accumulate data which argue for a direct interaction.

A first obvious, yet fundamental issue is to demonstrate that the miRNA and mRNA of interest are co-expressed in the same type of cells. Even if the miRNA and mRNA have been assayed in the samples of interest by previous high-throughput methods, it should be considered good scientific practice to support these results by additional techniques. This can be done by various standard biochemical methods, e.g., Northern blot or RT-qPCR for both miRNA and mRNA, and Western blot or enzyme-linked immunosorbent assay (ELISA) for the protein of interest. If research is focused on a particular tissue, *in situ* hybridization (to detect miRNAs or mRNAs) and immunofluorescence or immunohistochemistry (to detect proteins) can be regarded as gold standard to prove co-expression within the same type of cells [86]. As for candidate direct targets, the researcher should keep in mind that a considerable fraction of protein-coding genes is expressed as more than just a single transcript variant. Therefore, even if a particular gene might be predicted by all available target-prediction tools, it is still important to check if transcript variants exist that lack binding sites for the miRNA of interest (e.g., due to a shortened 3' UTR). RT-PCR experiments (followed by agarose gel electrophoresis) constitute a simple approach to demonstrate that, in the cells of interest, at least one transcript variant is expressed that is capable of being targeted by the respective miRNA.

The second important issue is to demonstrate the responsiveness of potential direct targets to modulation of the miRNA. Again, such responsiveness might have been assayed already by certain high-throughput methods; however, complementing techniques which reconfirm previous observations will greatly strengthen the scientific statements. Thus, gain-of-function experiments, e.g., by transfection of miRNA mimics or transduction of a vector that overexpresses the miRNA of interest, should be performed with the cells of interest to subsequently assay the potential direct target at mRNA and protein level, e.g., by RT-qPCR and Western blot. If effects can be observed, at least on mRNA level, already 24–48 h after the start of miRNA modulation, this is further supportive of a direct miRNA–mRNA interaction (as indirect effects, *i.e.*, cascades of other genes lying in between the miRNA and the mRNA, can be expected to need a longer time frame). Depending on the endogenous miRNA levels, it should be noted that gain-of-function studies can increase the miRNA abundance by several orders of magnitude and thus create a non-physiological situation [86]. Therefore, it is strongly recommended to perform also loss-of-function experiments, e.g., by transfection of antisense oligonucleotides (ASOs) or transduction of vectors expressing a corresponding miRNA sponge, to check whether the mRNA/protein of interest is elevated upon miRNA inhibition.

## 3. Validation of Direct miRNA–mRNA Interactions

When putative direct miRNA–mRNA interactions have been identified as described above, it is crucial to dissect which directly targeted mRNA follows as immediate next step in the pathway that the miRNA exerts its influence on. Therefore it is important to validate which candidate mRNA is actually physically bound by the miRNA, thus being a direct miRNA target. This permits ruling out an indirect effect or a signal cascade standing between the miRNA and the candidate target mRNA, confirming it as a direct miRNA–mRNA interaction.

### 3.1. Reporter Gene Assays

The current gold standard for plausibly proving direct interaction between miRNAs and their potential target mRNAs is the reporter gene assay. This usually involves the sequence harboring the MRE(s) (e.g., the 3' UTR) of the mRNA being cloned into a vector directly downstream of the reporter gene sequence (e.g., coding for a luciferase [87] or fluorescent protein [88]). This plasmid can then be used in gain- or loss-of-function experiments, by co-transfecting it together with a miRNA mimic or an ASO, respectively, as employed for miR-133 by Caré *et al.* [89]. In gain-of-function experiments, the transfected cell line of choice should optimally have low endogenous expression levels of the miRNA of interest [86], while in loss-of-function experiments, the endogenous miRNA expression levels should optimally be high. If the miRNA mimic directly interacts with the cloned MRE, expression of the reporter protein should be diminished and therefore, the output signal from cells transfected with this plasmid should be lower than that from cells transfected with an empty vector. *Vice versa*, co-transfection of an ASO that is specific for the miRNA of interest should in turn alleviate the effect and elevate reporter gene expression in case of genuine physical binding. As with target gain- and loss-of-function experiments, the effect of transfection should be controlled by transfection with a non-targeting control, and additionally by co-transfection with the empty vector and the MRE containing vector [90]. Before progressing to the next step, it is advisable to examine the levels of mature miRNA that were achieved by its overexpression or inhibition. Here, Northern blotting or RT-qPCR may be employed for overexpression as well as inhibition experiments, as long as one keeps in mind that depending on the inhibitor used, the detected miRNA levels might not reflect its actual cellular abundance due to formation of a miRNA-inhibitor complex that is not degraded. However, this can be alleviated by using a slightly modified RNA extraction protocol as shown by Torres *et al.* [91].

In order to prove that the miRNA of interest directly binds to a specific sequence, e.g., a distinct MRE, it is highly advisable to check whether this direct binding is depleted either by mutating this MRE or by inserting the MRE in inverse orientation. When an mRNA target sequence contains multiple MREs, site-directed mutagenesis can create specific seed match point mutations to identify those MRE(s) which have the strongest impact on the direct target mRNA regulation (e.g., shown by Xiao *et al.* [92]) [90,93,94]. Here, cold-fusion cloning is an option that has been used to create the required recombinant vectors faster than conventional cloning [95].

Even though this type of assay is the closest to mimicking *in vivo* interactions to date, one has to keep in mind that transfection with miRNA mimics may cause supra-physiological miRNA levels which may lead to artificial interactions with the potential target [86,96,97]. Additionally, the choice of cell line affects results by (i) endogenous microRNA levels and (ii) endogenous expression of co-factors which may promote or hinder miRNA–mRNA binding [86].

In the future, some of the limitations given above might be alleviated by implementation of an *in vivo* miRNA-target interaction assay for mammalian cells, analogous to the GFP sensor assay described by Schertel *et al.* in 2012 for *Drosophila melanogaster* [98]. A reported dual-luciferase assay for live tracking of miRNA dynamics in Zebrafish might one day be used to similar avail [99].

### 3.2. Electrophoretic Mobility Shift Assay (EMSA)

The electrophoretic mobility shift assay, which is routinely used to confirm binding of nucleic acids to proteins or other nucleic acids, has recently been adapted to conduct miRNA-target binding studies by Solé *et al.* 2013 [100]. In this publication, the authors propose the use of a miRNA mimic and the mRNA’s putative target sequence, e.g., the 3' UTR, as well as ASOs and mismatched negative controls. The miRNA in question is allowed to bind to the radiolabeled target mRNA probe in a polyacrylamide gel followed by drying, exposure, and analysis. Running the unbound miRNA and the target mRNA as controls is essential for electrophoretic mobility shift assay (EMSA), since the band(s) emerging in the control lane establish a baseline height for the probe in the gel. Binding of the probe to its target should shift the band(s) visibly from this baseline. Competition assays with unlabeled probes can further underscore the evidence of probe-target binding.

However, one critical weakness of EMSA for the scope of confirming direct miRNA-target interactions is its cell-free setting. This implies discounting the possible influence of additional physiological factors, such as RNA-binding proteins and the cell-specific abundance relationship to the miRNA and its target.

## 4. Deciphering the Biological Impact of Individual miRNA–mRNA Interactions

Once several targets that the miRNA directly binds to have been identified, it is of utmost importance to single out the target mRNA(s) that actually mediate the physiological effect that the miRNA has in the tissue/cell type of interest. This is necessary, since one miRNA can target hundreds of different mRNAs [28,36], which may not all be expressed in every kind of cell, and of which only some might be relevant to the distinct cell type and metabolic or signaling pathways investigated.

In contrast to the popular belief that miRNA–target interactions depend solely on sequence complementarity as well as expression levels of the miRNA and its target in the specified tissue, one has to additionally keep in mind that the functional effect of a miRNA can be influenced strongly by the expression signature of the tissue examined. For example, a class of SMAD proteins can affect essential miRNA maturation steps [101], or the miRNA–mRNA binding can be prevented by auxiliary RNA-binding proteins, thus alleviating the miRNA effect [102].

Currently, there are two distinct experimental approaches to convincingly show that a certain mRNA target mediates the miRNA effect you are interested in: phenocopy experiments, and disruption of miRNA–mRNA interactions.

### 4.1. Phenocopy Experiments

Multiple mRNAs may be identified as direct targets by one miRNA even in one single cell type. This begs the question: which of these target mRNAs is responsible for mediating the miRNA’s main effect? This issue can be tackled through the use of loss-of-function as well as gain-of-function experiments, as shown e.g., for miR-193b-365 [103]. The rationale here is that if selective down-regulation (loss-of-function) or elevation (gain-of-function) of the potential miRNA target yields a phenotype similar to upward/downward modulation of miRNA levels, this “miRNA-target axis” might be highly relevant for the investigated biological process.

Since miRNAs customarily exert their function by reducing expression levels of their target mRNAs, artificial knockdown of the target mRNA should produce a phenocopy of the miRNA over-expression effect. This shows conclusively that the observed effect is, out of several available and verified direct targets, mediated by the target mRNA of interest, as shown e.g., for miR-26 [90]. Target silencing is normally achieved by transfecting the cells with commercially available siRNA pools that target all transcript variants of the target mRNA, and thus modulating the target mRNA and protein levels [90]. However, if one or more of the target transcript variants expressed in the cell type of interest do not harbor miRNA binding sites, it is advisable to consider designing custom siRNAs which only target those transcript variants that bear a MRE. Otherwise, transcript variants might be affected that are not targeted by the miRNA under physiological circumstances which would lead to an overestimation of the miRNA effect on this target gene.

Loss-of function experiments may also be implemented by utilizing neutralizing antibodies in case of a secreted protein [104], or by pharmacological inhibitors of the target of interest [105]. As for the readout, the same techniques can be applied as were used for showing the miRNA’s effect in the first place (e.g., RT-qPCR for marker genes, Western Blotting, biochemical assays, histological stainings).

In some cases, the phenotype achieved by miRNA over-expression is the sum of its effect on more than one direct miRNA target. Then, one might not see a drastic and definite phenocopy effect upon silencing of one single target, but possibly a synergistic effect could emerge during co-silencing experiments of several direct target mRNAs [106].

On the contrary, successful target gene gain-of-function experiments aim to phenocopy the effects of miRNA inhibition. Consequently, this experimental approach is opposite to the target loss-of-function experiments illustrated above: Elevation of target gene levels is achieved by introducing target gene over-expression vectors [88,107], the addition of recombinantly expressed target proteins to the cell medium in case of secreted proteins [108], or by utilizing pharmacological agonists for protein activation.

An additional way to validate the necessity of a miRNA targeting a specific mRNA to achieve its effect is to conduct rescue experiments, wherein the miRNA is over-expressed in parallel with the putative target gene bearing a mutated MRE in its 3' UTR. This mutated MRE should ablate miRNA binding and down-regulation of target gene levels. *Vice versa*, one may also inhibit the miRNA of interest while simultaneously knocking down (antagonizing) the target mRNA to check whether this leads to a loss of the miRNA effect. In general, rescue experiments are an even stricter approach to underline the target gene’s role in mediating the miRNA’s impact on the cell.

If these loss- or gain-of-function experiments of the miRNA target gene phenocopy the effects of the corresponding miRNA modulation, respectively, the evaluated target gene can be considered as mediator of the miRNA effect, further underscoring this direct miRNA–mRNA interaction.

### 4.2. Disruption of Particular miRNA–mRNA Interactions

Another way of deciphering the biological impact of miRNA–target interactions are target protection experiments, where ASOs are designed that are complementary to a distinct MRE in a given miRNA target mRNA in order to deplete the miRNA-mediated repression of a particular direct target mRNA. This type of experiment has long been used to investigate miRNA-target specific effects in non-mammalian model systems [109,110].

The rationale here is that by allowing these ASOs to bind to the MREs in the mRNA of interest, they outcompete the miRNA and thus prohibit its effect on this specific target, while other possible targets of the miRNA in the same cell system remain unaffected. Thus, target protection experiments represent a valuable alternative to miRNA inhibition experiments, with the advantage of validating particular target mRNAs as mediators of the miRNA effect, thus producing a more specific effect that is able to better emulate physiological circumstances.

While morpholino ASOs are routinely used in these systems, they are not a viable option for mammalian cells [111]. Instead, Knauss *et al.* [111], have recently demonstrated a plasmid-based method for introducing target protectors into mouse cells, both *in vitro* and *in vivo*. The ASOs in question achieve the best protection effect when they are about 60 nucleotides long and harbor the MRE-complementary sequence in a central position, flanked by junk to achieve the appropriate length [111]. Furthermore, it is possible to use LNA-modified ASOs to achieve the same goal, which has been successfully employed *in vivo* in the investigation of the developing cortex in mice [107], as well as in murine lung endothelial tissue and the human HUVEC, HeLa and U373 MG cell lines [112,113].

However, it should be noted that it might be a challenge to find an appropriate sequence for the target mRNA protector, as it should have the characteristic to robustly bind and thereby block the MRE on one side, while on the other side it should not interfere with miRNA–target interactions at other MREs.

Another viable method for disruption of miRNA–target interactions is targeted genome editing. While in the past, zinc finger nucleases were used for this purpose, the bacterial adaptive immunity system, which uses clustered regularly interspaced short palindromic repeats (CRISPR) in association with the RNA-guided Cas9 DNase, has recently been adapted for just this application [114]. Out of three types of CRISPR/Cas systems in bacteria, only type II systems have the unique features of a trans-activating CRISPR RNA (tracrRNA) basepairing with CRISPR RNA (crRNA) to guide the Cas9 endonuclease to a target region in the genome. The Cas9 protein as well as the crRNA and tracrRNA are encoded in a single gene. In contrast to type I and type III systems, where other Cas endonucleases process the pre-crRNA transcript into a mature crRNA, in type II systems this maturation step of crRNA is achieved by the partially complementary tracrRNA, which is coded on the strand opposite the CRISPR locus, and which is essential to induce processing by a ds-RNA specific ribonuclease [114,115]. The resulting mature crRNA assembles into a complex with the Cas9 endonuclease, which is thus guided to its sequence-specific target region in the genome. This target region must partially basepair with the mature crRNA, not unlike the matching between the seed region of a miRNA and its target, and must contain a protospacer-adjacent motif (PAM) next to the complimentary region [114,116]. Most importantly though for application in higher eukaryotic systems, Jinek *et al.* revealed that it is possible and feasible to design hybrid RNAs which incorporate tracrRNA and crRNA into one single transcript, which is hence named guide RNA (gRNA). The gRNA interacts with the Cas9 endonuclease just like the tracrRNA–crRNA complex would, and guides it to cleave any double-stranded target DNA sequence of interest, anywhere in the genome [114].

This RNA-guided genome editing system allows for the exciting new possibility of targeted modification of both copies of a gene in higher eukaryote models, and has already been applied in zebrafish [117], mouse (*in vivo*) [118,119], and human cell culture (*in vitro*) [116,120]. Since it eliminates the flaws of RNAi of not facilitating a complete knockdown of a gene and of possible off-target results [121], the CRISPR-Cas9 system is highly promising for future applications to miRNA research: For example, one might be able to disrupt specific MREs within the target mRNA. This would provide a functional alternative to validating direct miRNA–target interactions with specific MREs via site-specific point mutations followed by miRNA modulation approaches or luciferase reporter assays, as described above.

## 5. Conclusions

**Scheme 1:**
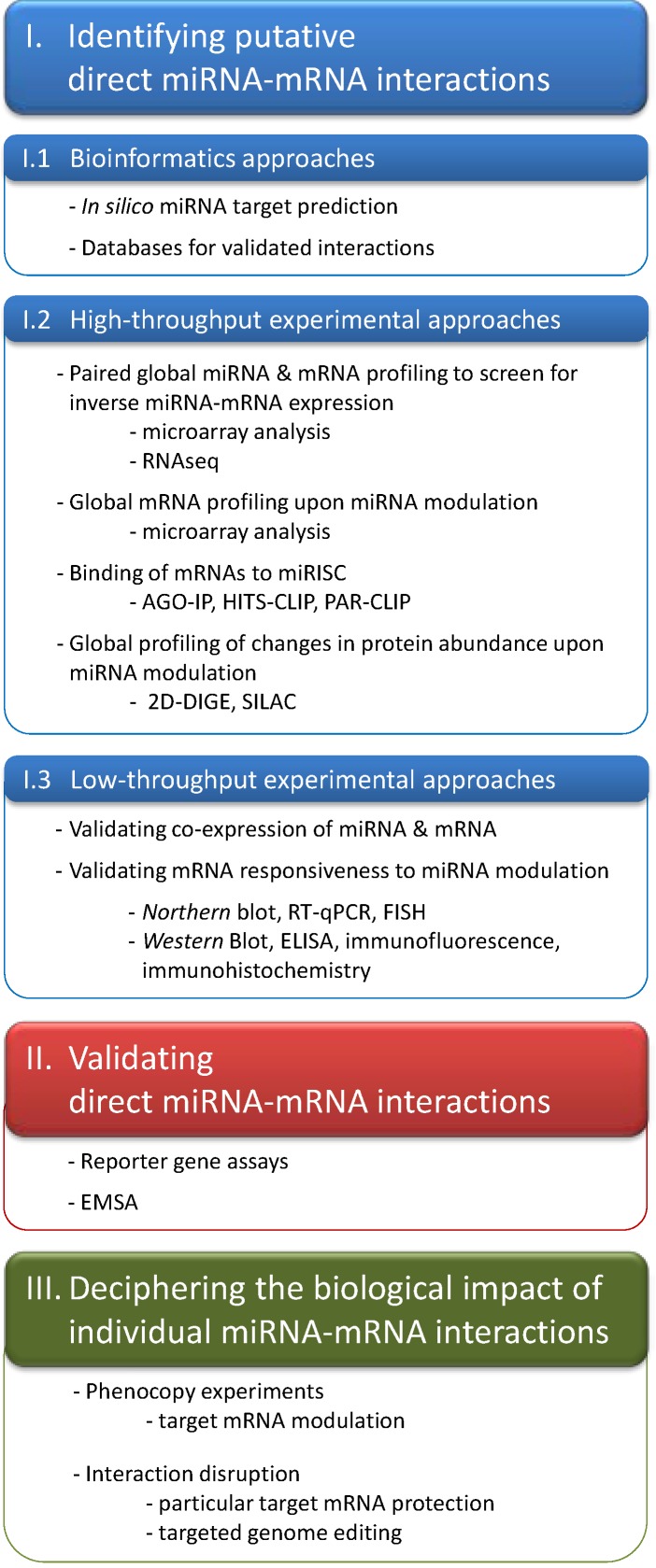
Workflow of methods to obtain biolotically meaningful microRNA targets.

This review provides a comprehensive workflow of methods for elucidating biologically meaningful miRNA targets, as summarized in Scheme 1. Starting with *in silico* analyses which are followed by high throughput approaches, the experimenter is guided through an assortment of methods to first identify mRNAs that are responsive to miRNA modulation, and then to validate direct miRNA–mRNA interactions. Finally, this review leads all the way to deciphering the biological impact of single miRNA–mRNA target pairs.

Since the discovery of miRNAs in 1993 [1] in *C. elegans*, waves of thousands of miRNAs have been identified across all borders of species, including 2500 entries for human alone in miRBase [122]. Individual members of this ncRNA class have been implicated in almost all biological processes as well as several diseases, including several types of cancer [88,123,124], Alzheimer’s disease [125], obesity [90,106,126,127,128] and type II diabetes [129,130]. As dozens to hundreds of mRNA targets have been postulated for each individual miRNA, the hunt for direct miRNA targets that mediate the miRNA effect is an essential step on the way from identifying a miRNA that creates a phenotype to linking it to a biological pathway and dissecting its implications in disease. The importance of this step becomes apparent when one considers that antagonizing or restoring miRNA function has already been recognized as an attractive intervention in several diseases. Applications range from the first miRNA inhibitor against hepatitis C virus, which is currently in clinical trial phase 2 [131], to miRNA mimics for cancer treatment which have just entered the clinic [132,133].

By presenting this guide to obtain biologically meaningful miRNA targets through the jungle of available methods, we hope to aid researchers aiming to illuminate further miRNA–target interactions which might be relevant to human biology and disease.
